# Supplementary material for: Motif-Centered Analyses Reveal Universal and Tissue-Specific Mutagenic Mechanisms Operating in the Human Body
Source: bioRxiv. 2026 May 10:2025.10.07.681015. Preprint. [Version 4] doi: 10.1101/2025.10.07.681015 (PMC12934804; doi:10.1101/2025.10.07.681015)
Supplement: Supplement 1 [file media-1.zip › Supplementary_Data_S1.zip!/Supplemental_Data_S1/Dermis/Dermis_feature_A.pdf]

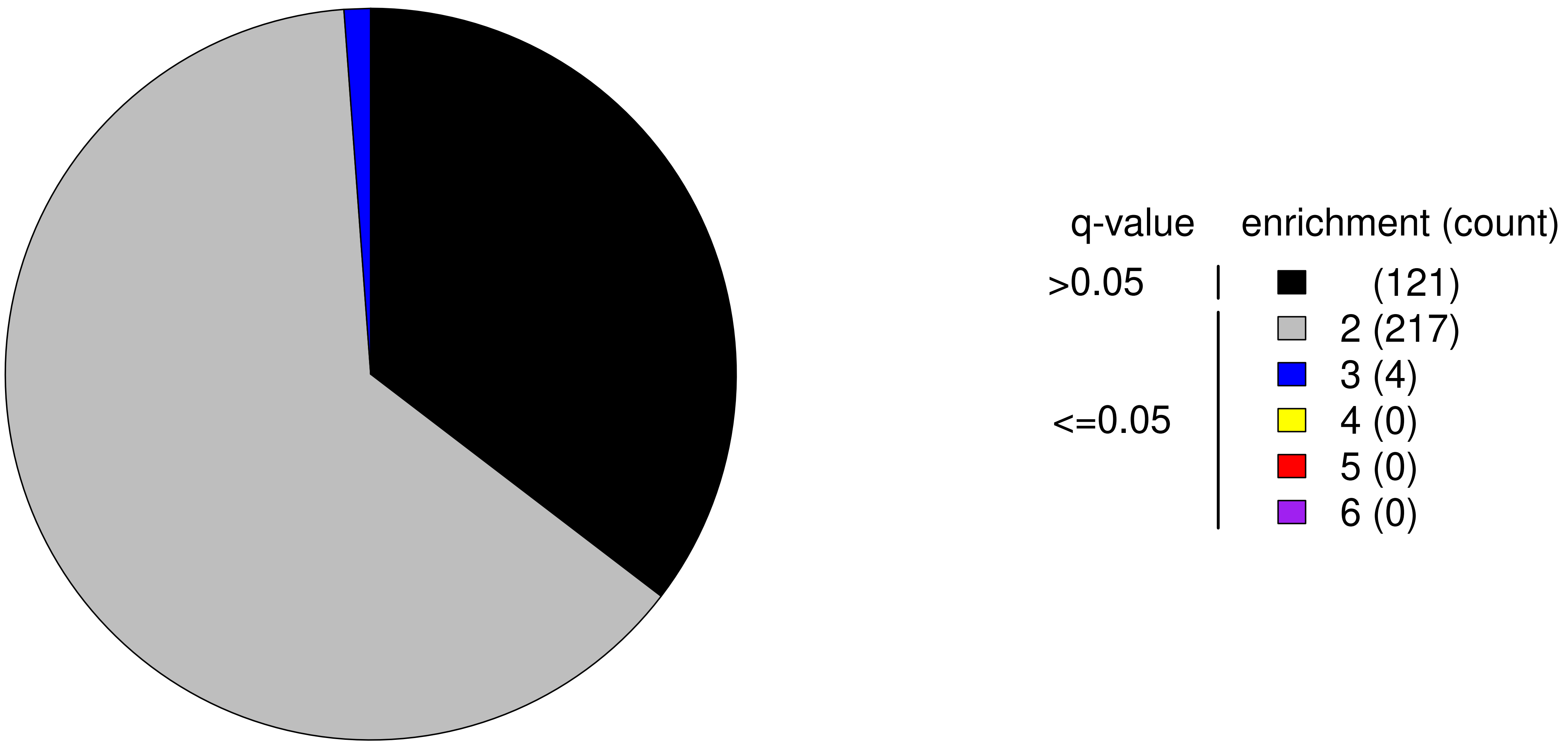
q-value
enrichment (count)
>0.05
(121)
2 (217)
<=0.05
3 (4)
4 (0)
5 (0)
6 (0)
