# Supplementary material for: Motif-Centered Analyses Reveal Universal and Tissue-Specific Mutagenic Mechanisms Operating in the Human Body
Source: bioRxiv. 2026 May 10:2025.10.07.681015. Preprint. [Version 4] doi: 10.1101/2025.10.07.681015 (PMC12934804; doi:10.1101/2025.10.07.681015)
Supplement: Supplement 1 [file media-1.zip › Supplementary_Data_S1.zip!/Supplemental_Data_S1/Dermis/Dermis_feature_B.pdf]

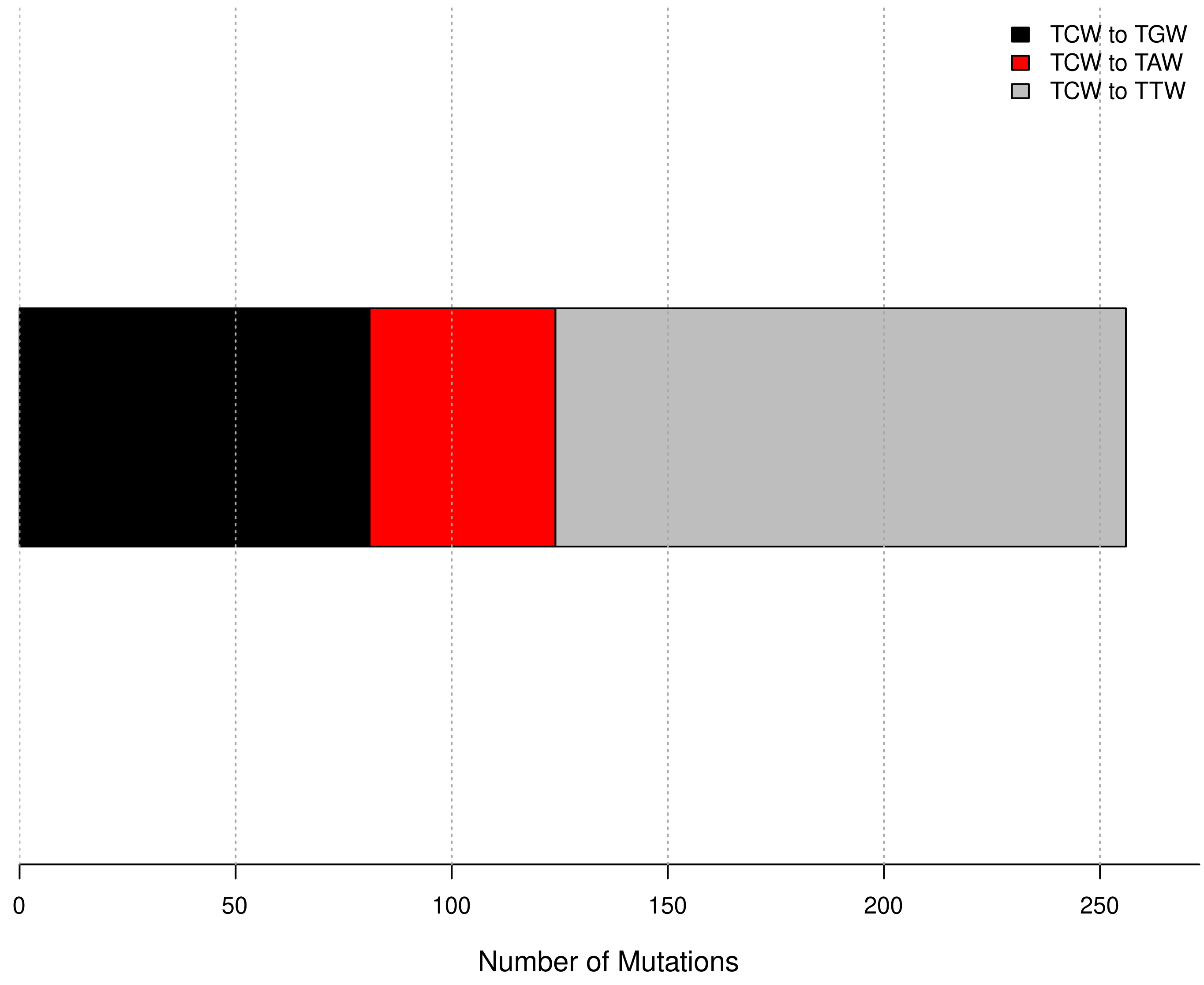
TCW to TGW
TCW to TAW
TCW to TTW
0
50
100
150
200
250
Number of Mutations
