# Supplementary material for: Motif-Centered Analyses Reveal Universal and Tissue-Specific Mutagenic Mechanisms Operating in the Human Body
Source: bioRxiv. 2026 May 10:2025.10.07.681015. Preprint. [Version 4] doi: 10.1101/2025.10.07.681015 (PMC12934804; doi:10.1101/2025.10.07.681015)
Supplement: Supplement 1 [file media-1.zip › Supplementary_Data_S1.zip!/Supplemental_Data_S1/Dermis/Dermis_feature_C.pdf]

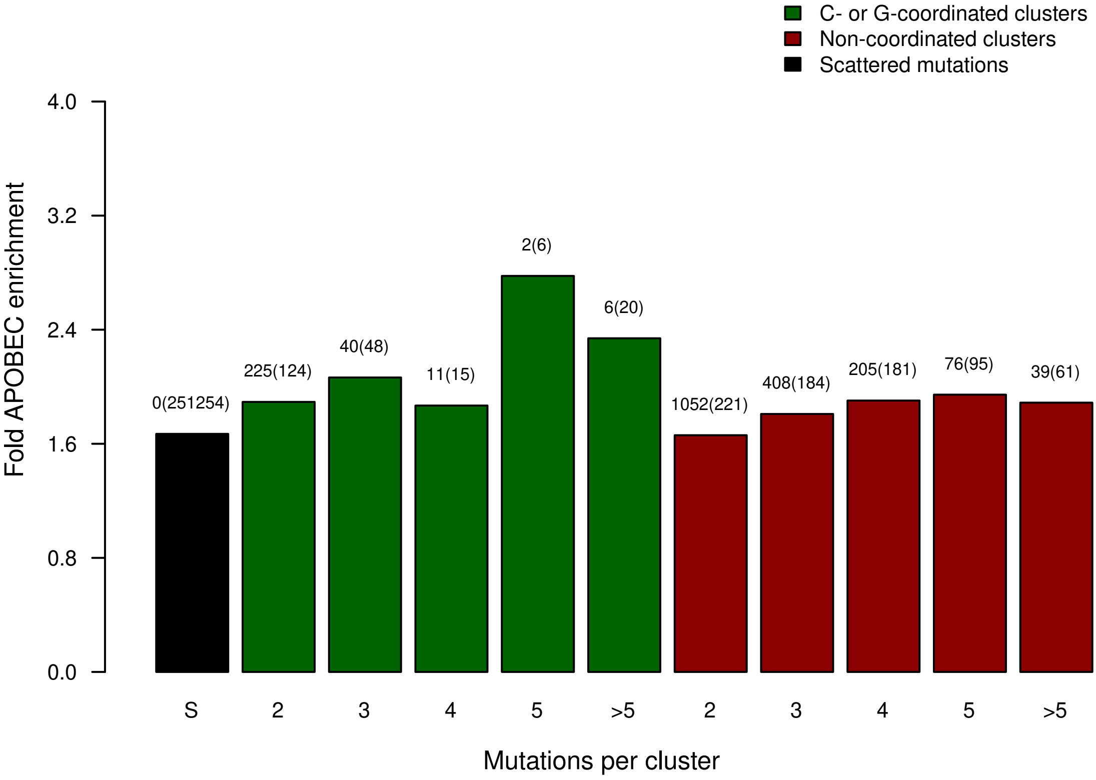
C- or G-coordinated clusters
Non-coordinated clusters
Scattered mutations
Fold APOBEC enrichment
4.0
3.2
2.4
1.6
0.8
0.0
0(251254)
225(124)
40(48)
11(15)
2(6)
6(20)
1052(221)
408(184)
205(181)
76(95)
39(61)
S
2
3
4
5
>5
2
3
4
5
>5
Mutations per cluster
