# Supplementary material for: Motif-Centered Analyses Reveal Universal and Tissue-Specific Mutagenic Mechanisms Operating in the Human Body
Source: bioRxiv. 2026 Mar 27:2025.10.07.681015. Preprint. [Version 3] doi: 10.1101/2025.10.07.681015 (PMC12934804; doi:10.1101/2025.10.07.681015)
Supplement: Supplementary file 1 [file NIHPP2025.10.07.681015v3-supplement-2.pdf]

bioRxiv preprint doi: https://doi.org/10.1101/2025.10.07.681015; this version posted March 27, 2026. The copyright holder for this preprint (which was not certified by peer review) is the author/funder. This article is a US Government work. It is not subject to copyright under 17 USC 105 and is also made available for use under a CC0 license.

Supplementary information

Supplementary figures

Supplementary Figure S1. Somatic mutation spectra and contribution of COSMIC signatures in diseased tissues. **A**. Median counts for each base substitution including reverse complements are shown in descending order for diseased WGS and WES samples. **B.** Relative contributions of published COSMIC signatures in the mutation profiles of WGS diseased tissues. All source data are available in Supplementary Table S3.

Supplementary Figure S2. SBS signatures and mutational motifs in exome-sequenced samples. **A.** Relative contributions of published COSMIC signatures in the mutation profiles of WES samples are shown for healthy and diseased tissues. **B-C**. The percentages of samples with zero and non-zero MEML counts for the 11 known mutational motifs calculated for WES samples in healthy **(B)** and diseased **(C)** tissues. Source data are available in Supplementary Tables S3 and S4.

Supplementary Figure S3. COSMIC signature profiles and correlating motifs. Plots displaying mutation profiles for GRCh38 with assigned etiologies were downloaded from COSMIC website and shown for signatures **(A)** SBS1, SBS5, SBS16, **(B)** SBS7a, SBS7b, SBS2, and SBS13. Motifs correlating with the signatures are indicated on each plot with vertical bars under corresponding motif peaks in signature spectra. Bar colors indicate individual motifs.

Supplementary Figure S4. Analysis of aTn→aCn sub-motifs to detect true presence of aTn motif in healthy tissues. Scatter plots showing aTn motif MEML plotted against aTr motif MEML in WGS samples across different tissues. Blue line indicates line of best fit from a simple linear model.

Supplementary Figure S5. Analyses of aTn motif and sub-motifs in samples with non-zero MEML values. A. Correlation matrix showing Spearman correlation between aTn→aCn MEML and aTr→aCr MEML excluding either MEML =0 to identify true aTn mutational motif. Grey circles indicate P value >0.05 after correction for multiple hypothesis using Benjamini-Hochberg method within each sequencing group (WGS or WES). No circles indicate insufficient samples with MEML>0 for a tissue type to perform correlation analyses. **B.** Correlation matrix showing donor age versus mean donor MEML of aTn and aTr motifs excluding either MEML=0. Spearman

bioRxiv preprint doi: https://doi.org/10.1101/2025.10.07.681015; this version posted March 27, 2026. The copyright holder for this preprint (which was not certified by peer review) is the author/funder. This article is a US Government work. It is not subject to copyright under 17 USC 105 and is also made available for use under a CC0 license.

correlations were performed between donor age and mean MEML count of all samples from an individual donor for the indicated tissues and mutational motifs. Only tissues with sufficient number of donors to support age-correlation analyses are shown. Grey circles indicate P value >0.05 after correction for multiple hypothesis using Benjamini-Hochberg method for a given motif within each sequencing group (WGS or WES). No circles indicate insufficient individual donors with mean MEML>0 for to perform correlation analyses. All source data and statistical analyses can be found in Supplementary Table S5.

Supplementary Figure S6. Analysis of nCg→nTg sub-motifs and their correlation with each other in healthy tissues. A. Scatter plot showing nCg→nTg MEML plotted against MEML of sub-motif rCg→rTg, the component of nCg non-overlapping to UV-motif yCn, in WGS samples across different tissues. Blue line indicates line of best fit from a simple linear model. **B.** Correlation matrix showing Spearman correlation of nCg→nTg with sub-motifs rCg→rTg (UV non-overlapping) and yCg→yTg (UV overlapping), excluding either MEML =0. Grey circles indicate P value >0.05 after correction for multiple hypothesis using Benjamini-Hochberg method within each sequencing group (WGS or WES). No circles indicate insufficient samples with MEML>0 for a tissue type to perform correlation analyses. C. Scatter plot showing nCg→nTg MEML plotted against MEML of yCh→yTh, the component of UV-motif yCn non-overlapping to nCg, in WGS samples of dermis and epidermis. Blue line indicates line of best fit from a simple linear model. All source data and statistical analyses can be found in Supplementary Table S6.

Supplementary Figure S7. Analysis of nCg→nTg sub-motifs and their correlations with donor age in healthy tissues. A. Scatter plot showing nCg→nTg MEML plotted against MEML of sub-motif yCg→yTg, the component of nCg overlapping to UV-motif yCn, in WGS samples across different tissues. Blue line indicates line of best fit from a simple linear model. **B.** Correlation matrix showing Spearman correlation of donor age with donor-specific mean MEML value for nCg→nTg and sub-motifs rCg→rTg (UV non-overlapping) and yCg→yTg (UV overlapping), excluding either MEML =0. Grey circles indicate P value >0.05 after correction for multiple hypothesis using Benjamini-Hochberg method within each sequencing group (WGS or WES). No circles indicate insufficient samples with MEML>0 for a tissue type to perform correlation analyses. All source data and statistical analyses can be found in Supplementary Table S6.

Supplementary Figure S8: Tissues with multiple features indicating APOBEC mutagenesis. A. Fold-enrichment of APOBEC mutagenesis motif over the expected occurrence of random mutagenesis in the indicated tissues. Samples are categorized into color coded bins based on their FDR corrected q-values (Benjamini-Hochberg) and fold enrichment values. Fold enrichment bin sizes are increments of 1 unit. All samples displaying a q-value >0.05 are placed in one bin (black) regardless of the fold APOBEC enrichment in the sample. The maximum fold enrichment for each bin is indicated in the figure legend, with the number of samples in its category shown in parentheses. **B.** The number of mutations categorized into three possible types of substitutions at the tCw motif (complementary mutations included) within C- or G-coordinated clusters in the indicated tissues. **C.** Fold enrichment of the APOBEC motif mutations in clusters of different sizes of two categories – C- or G-coordinated, non-coordinated, as well as in non-clustered (scattered) mutations in the indicated tissues. Values above the bars display the total number of mutation clusters in each class and in parentheses the total numbers of APOBEC motif mutations in a category. **D.** Enrichment of ytCa (APOBEC3A-like) motif and rtCa (APOBEC3B-like) motif among genome-wide, scattered, clustered, and G- or C- coordinated clustered mutations in the indicated tissues. The blue dashed vertical line indicates level of no enrichment. Where enrichment of both ytCa and rtCa motifs were statistically significant (Fisher's Exact P-value ≤0.05), Breslow-Day test of homogeneity was performed to compare the odds ratios of the two motif enrichments. Asterisks indicate significant difference at P-value ≤0.05. Source data for plots **A**, **B**, and **C** are available in

bioRxiv preprint doi: https://doi.org/10.1101/2025.10.07.681015; this version posted March 27, 2026. The copyright holder for this preprint (which was not certified by peer review) is the author/funder. This article is a US Government work. It is not subject to copyright under 17 USC 105 and is also made available for use under a CC0 license.

Supplementary Data S1. Source data and statistical analyses for ytCa and rtCa enrichment **(D)** can be found in Supplementary Table S8e and S8f.

Supplementary Figure S9. Multivariable regression analyses of aTn and nCg motifs in non-cancer diseases. β-coefficients from multivariable regression analyses using donor age and disease status as predictor variables are plotted for brain **(A)**, liver **(B)**, colon **(C)**, lung **(D)**, and small intestine **(E)**. *P value ≤ 0.05, **P value ≤ 0.01, ***P value ≤ 0.001. Source data are available in Supplementary Tables S9c and S10d.

Supplementary Figure S10. Analyses of sub-motifs to detect true spontaneous meCpG cytosine deamination-mediated nCg→nTg in diseased tissues. Correlation matrix showing Spearman correlation of nCg→nTg with sub-motifs rCg→rTg (UV non-overlapping) and yCg→yTg (UV overlapping), including MEML = 0 **(A)** and excluding pairs with either member's MEML =0 **(B)** in different disease categories. Grey circles indicate P value >0.05 after correction for multiple hypothesis using Benjamini-Hochberg method within each sequencing group (WGS or WES). No circles indicate insufficient samples with MEML>0 for a tissue type to perform correlation analyses. Source data are available in Supplementary Table S10b.

Supplementary Tables

Supplementary Table S1: Origins of mutation calls for samples analyzed in this study.

Supplementary Table S2: Sub-motifs of knowledge-based motifs with overlapping and non-overlapping components.

Supplementary Table S3: Somatic mutation spectra and COSMIC SBS signatures in normal cells. Contains numeric values underlying Figure 1, Supplementary Figures S1, S2a.

Supplementary Table S4: Prevalence of knowledge-based motifs in normal tissues. Contains numeric values underlying Figures 2, 3, Supplementary Figure S2b.

Supplementary Table S5: aTn mutational motif in normal healthy cells. Contains numeric values underlying Figure 4, Supplementary Figures S4, S5.

Supplementary Table S6: nCg mutational motif in normal healthy cells. Contains numeric values underlying Figure 5, Supplementary Figures S6, S7.

Supplementary Table S7: UV mutational motif in normal cells. Contains numeric values underlying Figure 6.

Supplementary Table S8: APOBEC mutational motif in healthy normal tissues. Contains numeric values underlying Figure 7, Supplementary Figure S8.

Supplementary Table S9: Prevalence of aTn motif in diseased tissues. Contains numeric values underlying Figure 8, Supplementary Figure S9.

Supplementary Table S10: Prevalence of nCg mutational motif in diseased samples. Contains numeric values underlying Figure 9, Supplementary Figures S9, S10.

Supplementary Data

Supplementary Data S1: Raw output of P-MACD motif and cluster analyses to detect APOBEC mutagenesis. Contains numeric values underlying Supplementary Figure S8.

Figure S1

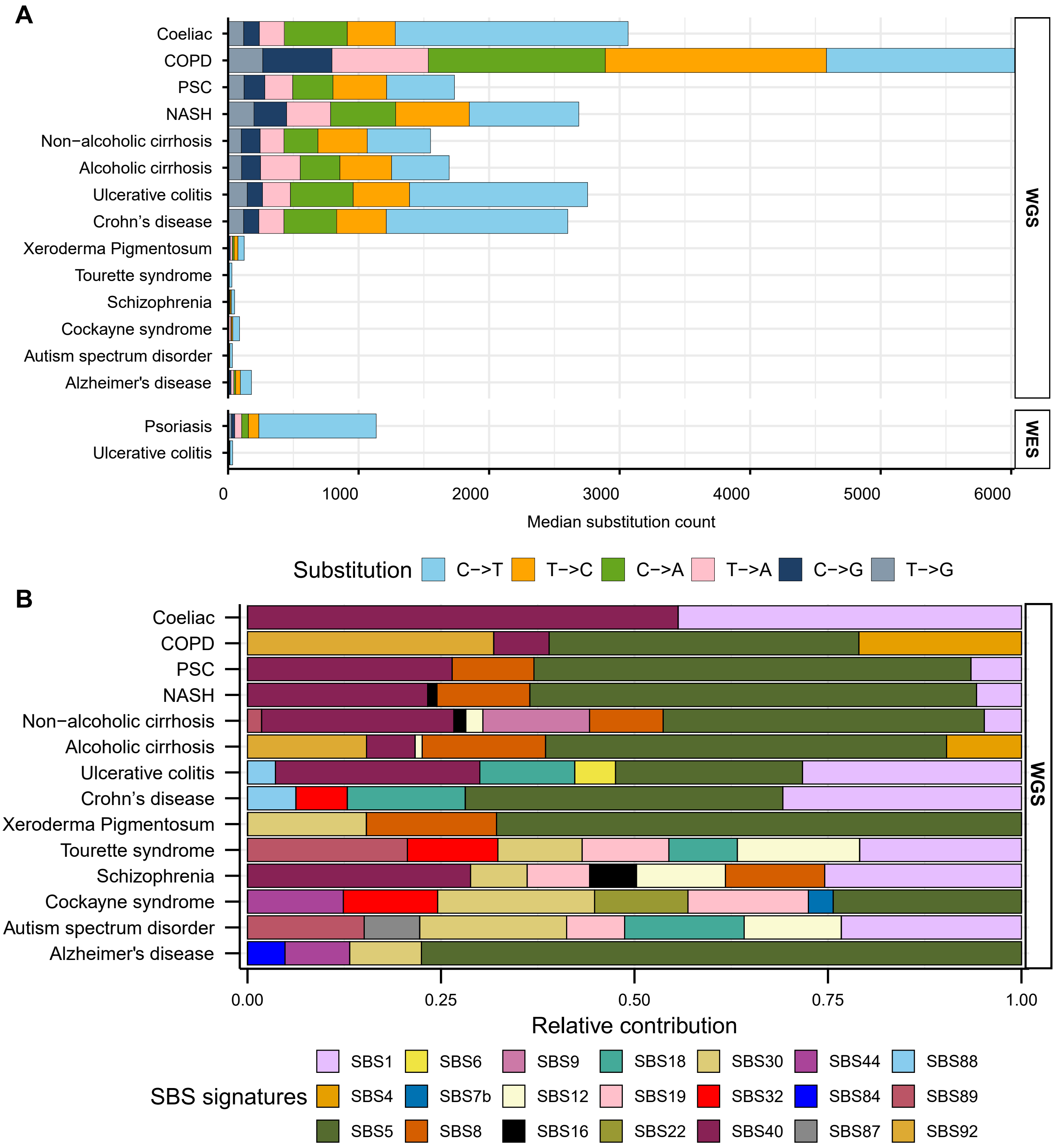
A
Coeliac
COPD
PSC
NASH
Non−alcoholic cirrhosis
Alcoholic cirrhosis
Ulcerative colitis
Crohn’s disease
Xeroderma Pigmentosum
Tourette syndrome
Schizophrenia
Cockayne syndrome
Autism spectrum disorder
Alzheimer's disease
Psoriasis
Ulcerative colitis
WGS
WES
0
1000
2000
3000
4000
5000
6000
Median substitution count
Substitution
C−>T
T−>C
C−>A
T−>A
C−>G
T−>G
B
Coeliac
COPD
PSC
NASH
Non−alcoholic cirrhosis
Alcoholic cirrhosis
Ulcerative colitis
Crohn’s disease
Xeroderma Pigmentosum
Tourette syndrome
Schizophrenia
Cockayne syndrome
Autism spectrum disorder
Alzheimer's disease
WGS
0.00
0.25
0.50
0.75
1.00
Relative contribution
SBS signatures
SBS1
SBS6
SBS9
SBS18
SBS30
SBS44
SBS88
SBS4
SBS7b
SBS12
SBS19
SBS32
SBS84
SBS89
SBS5
SBS8
SBS16
SBS22
SBS40
SBS87
SBS92

Figure S2

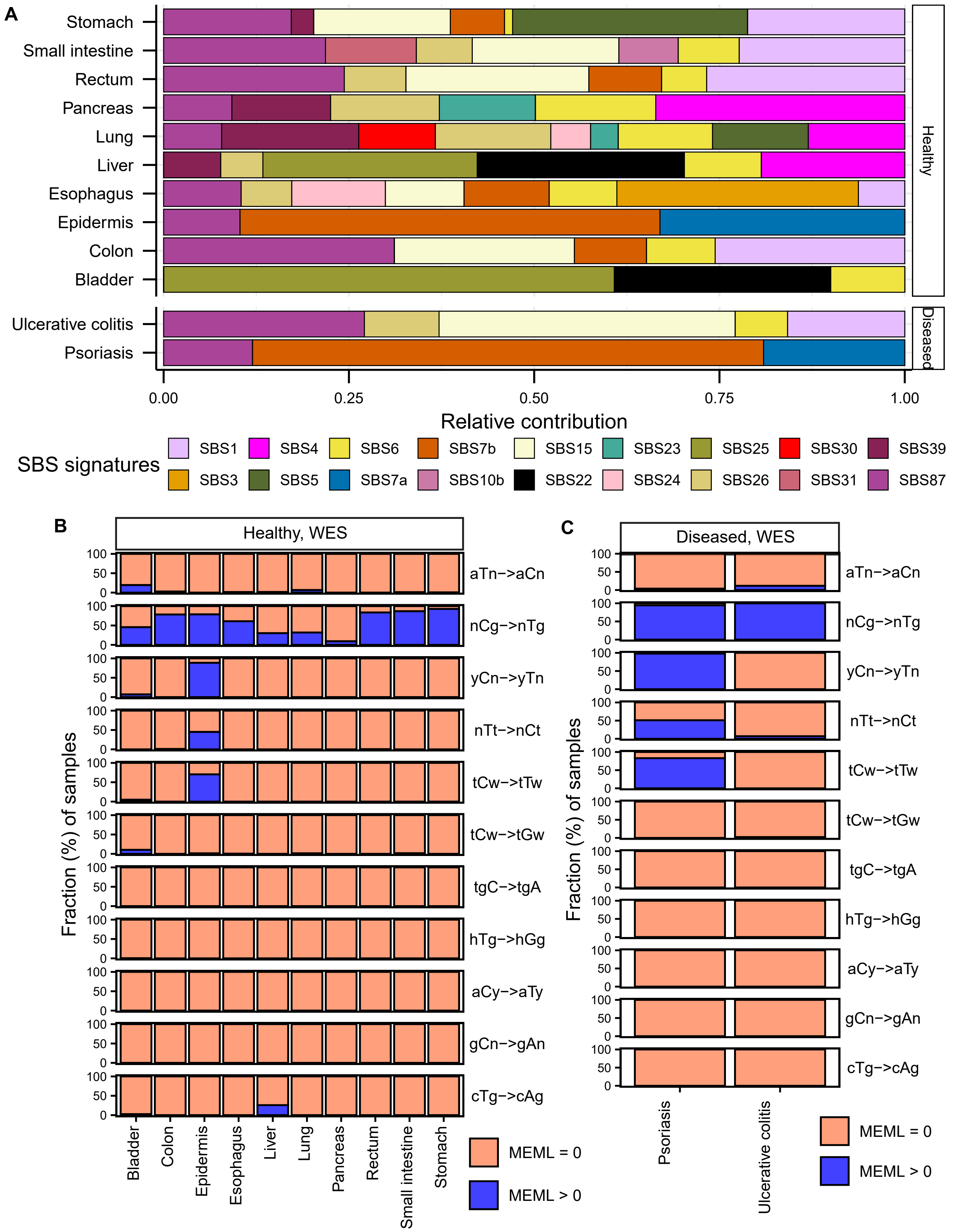
A
Stomach
Small intestine
Rectum
Pancreas
Lung
Liver
Esophagus
Epidermis
Colon
Bladder
Ulcerative colitis
Psoriasis
Healthy
Diseased
0.00
0.25
0.50
0.75
1.00
Relative contribution
SBS signatures
SBS1
SBS4
SBS6
SBS7b
SBS15
SBS23
SBS25
SBS30
SBS39
SBS3
SBS5
SBS7a
SBS10b
SBS22
SBS24
SBS26
SBS31
SBS87
B
Healthy, WES
Fraction (%) of samples
100
50
0
aTn−>aCn
nCg−>nTg
yCn−>yTn
nTt−>nCt
tCw−>tTw
tCw−>tGw
tgC−>tgA
hTg−>hGg
aCy−>aTy
gCn−>gAn
cTg−>cAg
Bladder
Colon
Epidermis
Esophagus
Liver
Lung
Pancreas
Rectum
Small intestine
Stomach
MEML = 0
MEML > 0
C
Diseased, WES
Psoriasis
Ulcerative colitis

Figure S3A

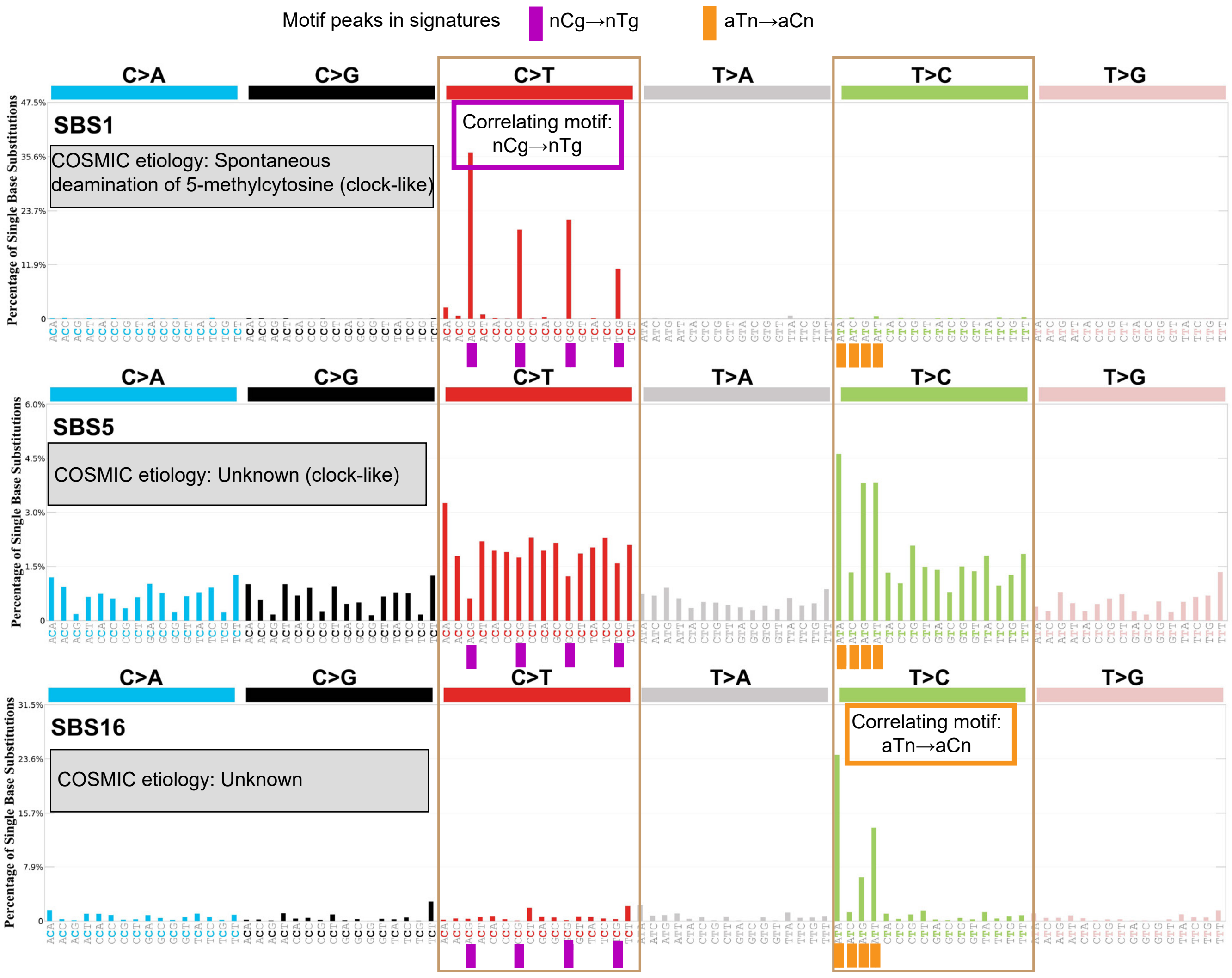

Motif peaks in signatures
nCg→nTg
aTn→aCn
C>A
C>G
C>T
T>A
T>C
T>G
SBS1
COSMIC etiology: Spontaneous deamination of 5-methylcytosine (clock-like)
Correlating motif: nCg→nTg
Percentage of Single Base Substitutions
47.5%
35.6%
23.7%
11.9%
0
SBS5
COSMIC etiology: Unknown (clock-like)
6.0%
4.5%
3.0%
1.5%
0
SBS16
COSMIC etiology: Unknown
Correlating motif: aTn→aCn
31.5%
23.6%
15.7%
7.9%
0

Figure S3B

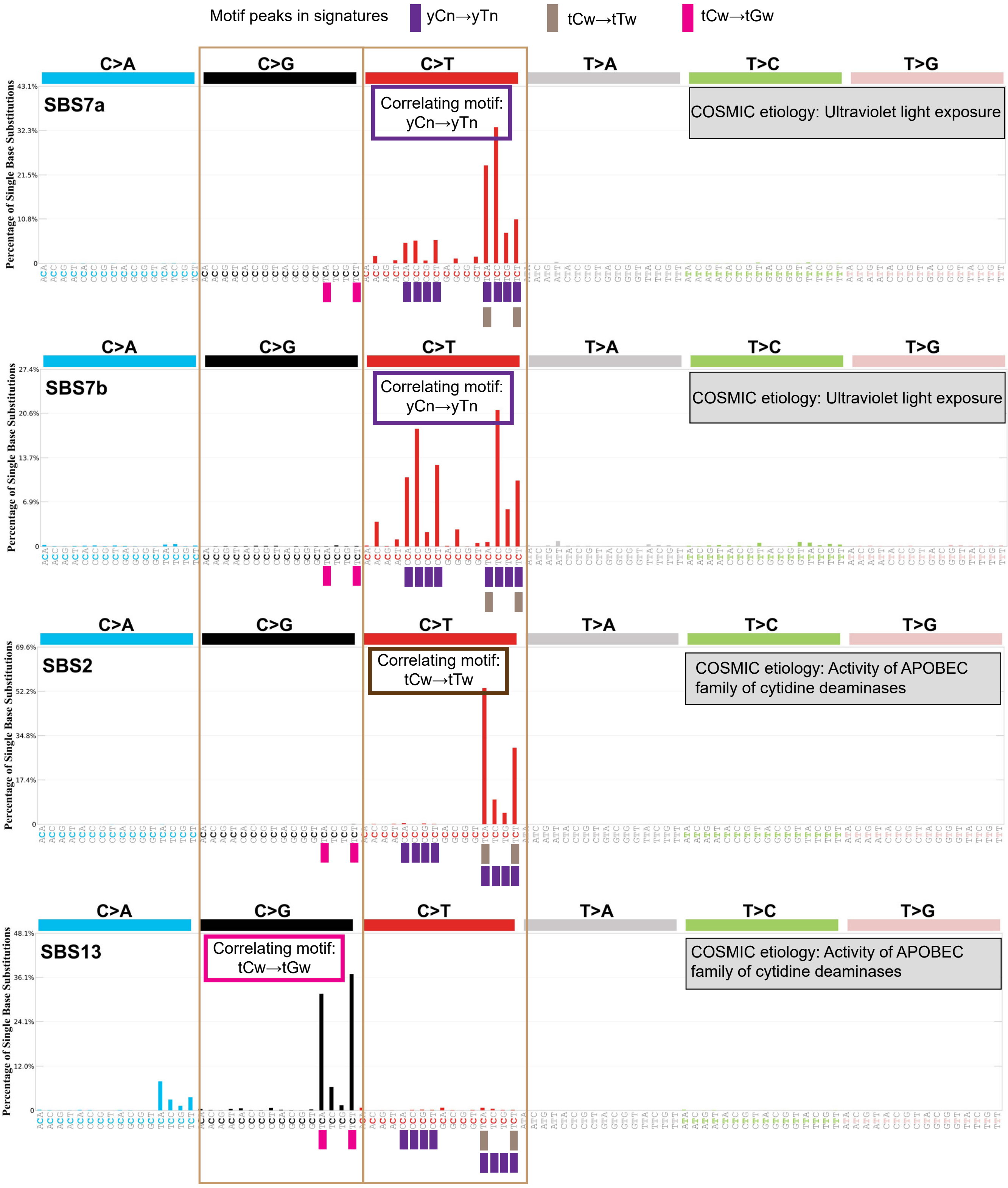
Motif peaks in signatures
yCn→yTn
tCw→tTw
tCw→tGw
C>A
C>G
C>T
T>A
T>C
T>G
Percentage of Single Base Substitutions
SBS7a
Correlating motif: yCn→yTn
COSMIC etiology: Ultraviolet light exposure
SBS7b
Correlating motif: yCn→yTn
COSMIC etiology: Ultraviolet light exposure
SBS2
Correlating motif: tCw→tTw
COSMIC etiology: Activity of APOBEC family of cytidine deaminases
SBS13
Correlating motif: tCw→tGw
COSMIC etiology: Activity of APOBEC family of cytidine deaminases

Figure S4

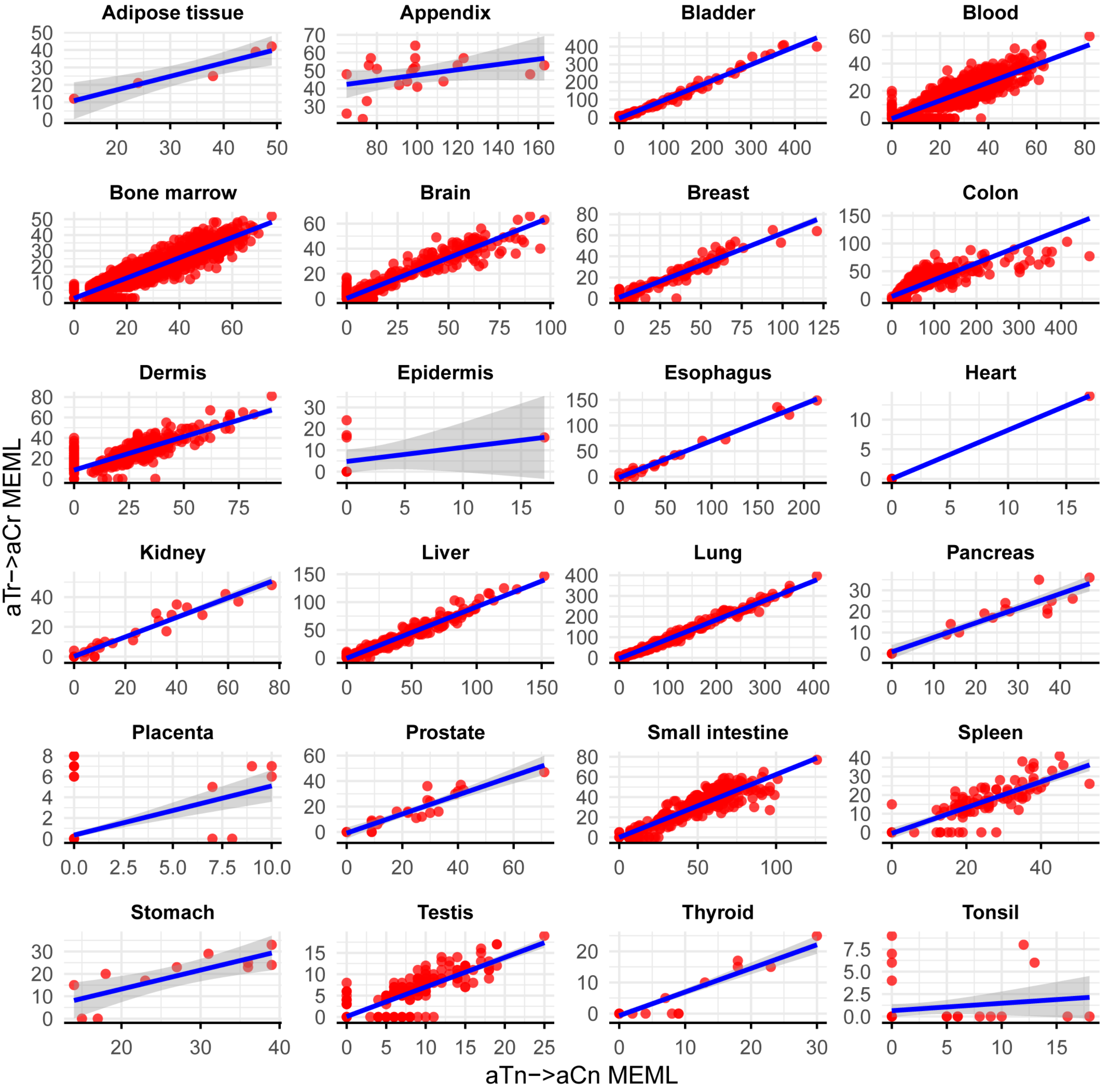

Adipose tissue
Appendix
Bladder
Blood
Bone marrow
Brain
Breast
Colon
Dermis
Epidermis
Esophagus
Heart
Kidney
Liver
Lung
Pancreas
Placenta
Prostate
Small intestine
Spleen
Stomach
Testis
Thyroid
Tonsil
aTr−>aCr MEML
aTn−>aCn MEML

Figure S5

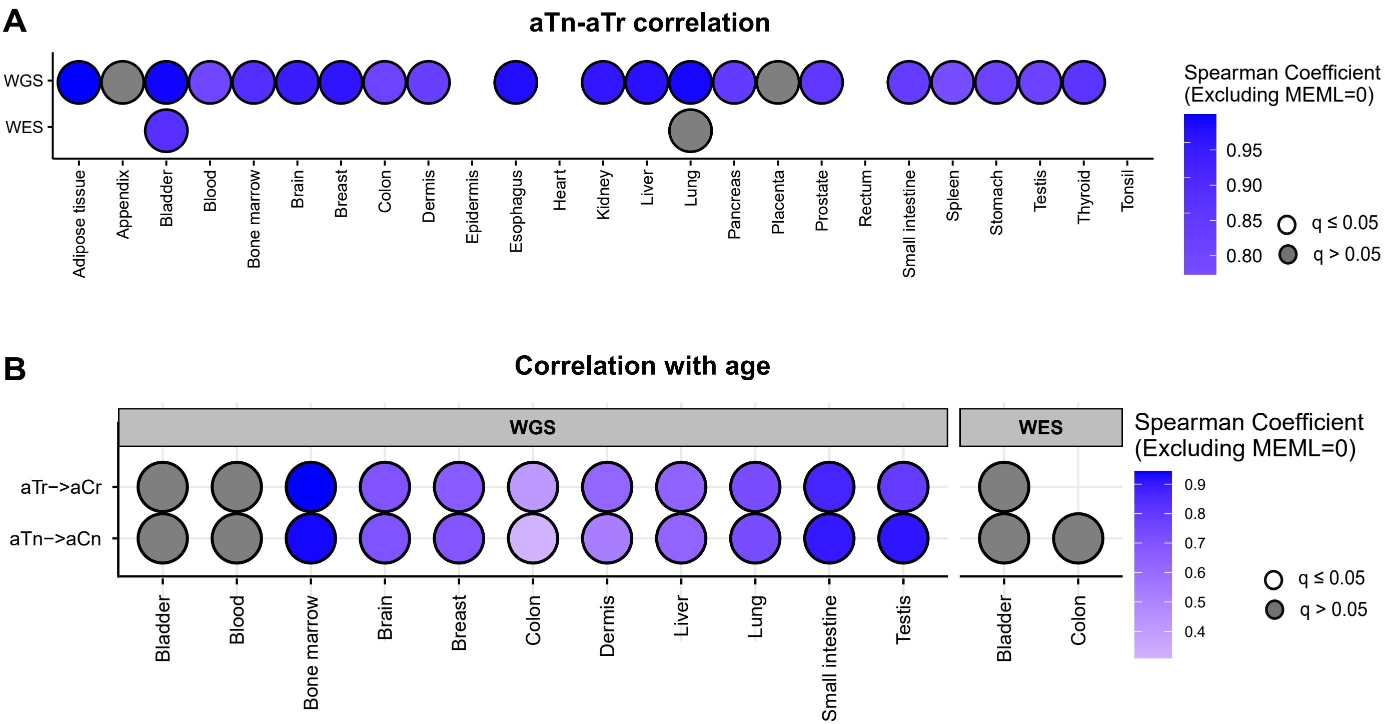
A
aTn-aTr correlation
WGS
WES
Adipose tissue
Appendix
Bladder
Blood
Bone marrow
Brain
Breast
Colon
Dermis
Epidermis
Esophagus
Heart
Kidney
Liver
Lung
Pancreas
Placenta
Prostate
Rectum
Small intestine
Spleen
Stomach
Testis
Thyroid
Tonsil
Spearman Coefficient
(Excluding MEML=0)
0.95
0.90
0.85
0.80
q ≤ 0.05
q > 0.05
B
Correlation with age
WGS
WES
aTr−>aCr
aTn−>aCn
Bladder
Blood
Bone marrow
Brain
Breast
Colon
Dermis
Liver
Lung
Small intestine
Testis
Bladder
Colon
Spearman Coefficient
(Excluding MEML=0)
0.9
0.8
0.7
0.6
0.5
0.4
q ≤ 0.05
q > 0.05

Figure S6

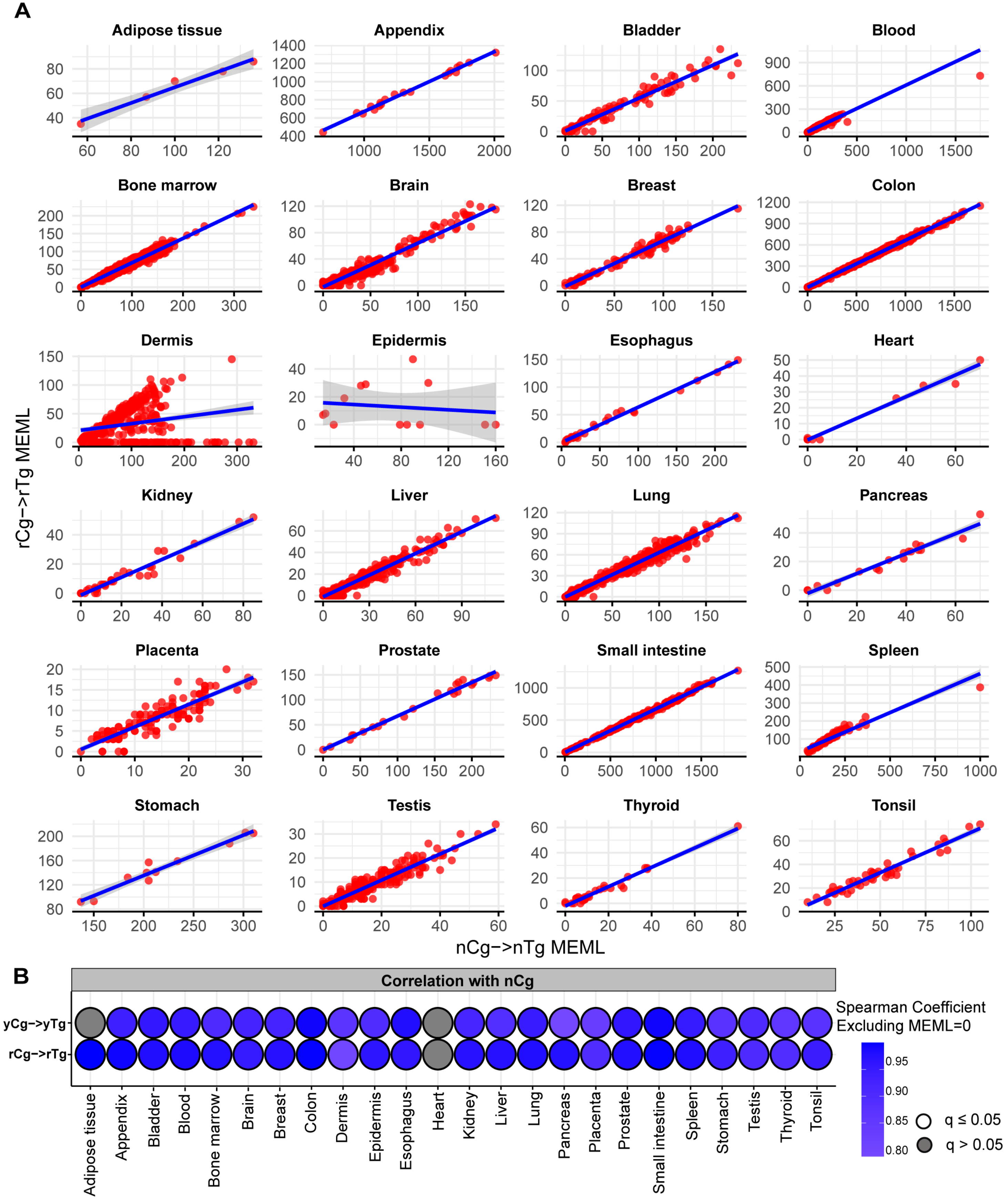
A
Adipose tissue
Appendix
Bladder
Blood
Bone marrow
Brain
Breast
Colon
Dermis
Epidermis
Esophagus
Heart
Kidney
Liver
Lung
Pancreas
Placenta
Prostate
Small intestine
Spleen
Stomach
Testis
Thyroid
Tonsil
rCg−>rTg MEML
nCg−>nTg MEML
B
Correlation with nCg
yCg−>yTg
rCg−>rTg
Adipose tissue
Appendix
Bladder
Blood
Bone marrow
Brain
Breast
Colon
Dermis
Epidermis
Esophagus
Heart
Kidney
Liver
Lung
Pancreas
Placenta
Prostate
Small intestine
Spleen
Stomach
Testis
Thyroid
Tonsil
Spearman Coefficient
Excluding MEML=0
0.95
0.90
0.85
0.80
q ≤ 0.05
q > 0.05

bioRxiv preprint doi: https://doi.org/10.1101/2025.10.07.681015; this version posted March 27, 2026. The copyright holder for this preprint (which was not certified by peer review) is the author/funder. This article is a US Government work. It is not subject to copyright under 17 USC 105 and is also made available for use under a CC0 license.

Figure S7

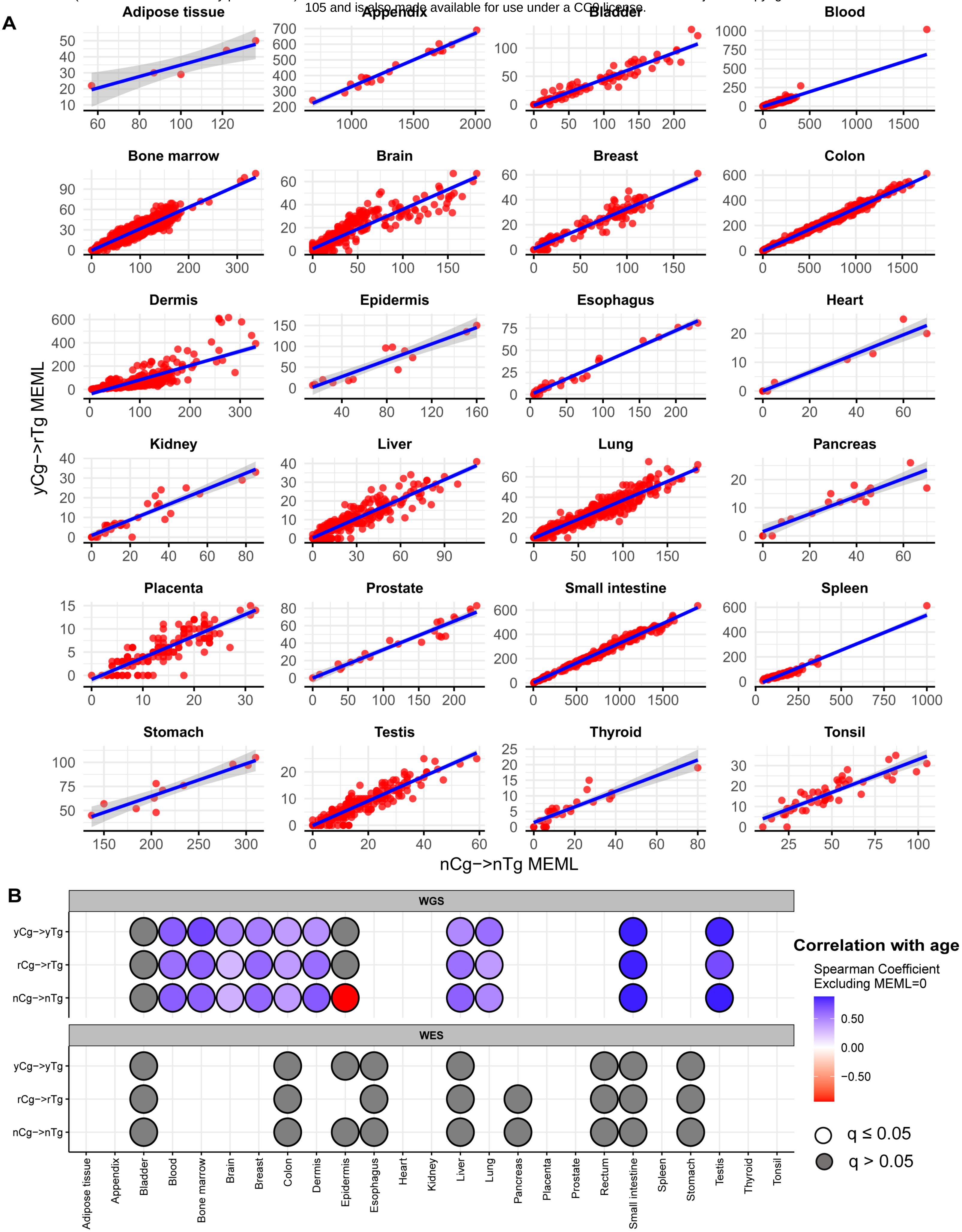

Figure S8

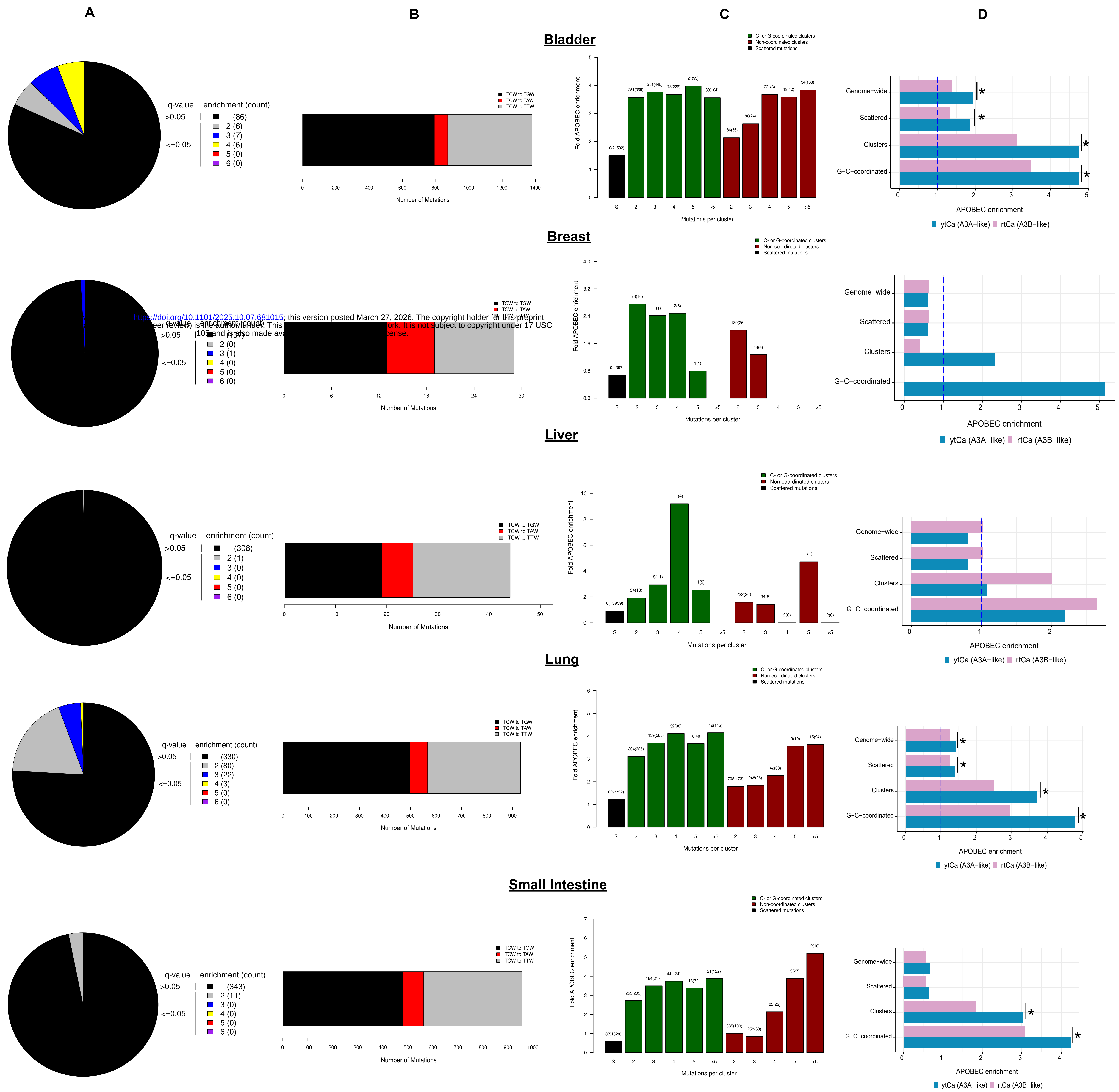

https://doi.org/10.1101/2025.10.07.681015; this version posted March 27, 2026. The copyright holder for this preprint
(which was not certified by peer review) is the author/funder. This [illegible] work. It is not subject to copyright under 17 USC
105 and is also made ava[illegible] license.

Figure S9

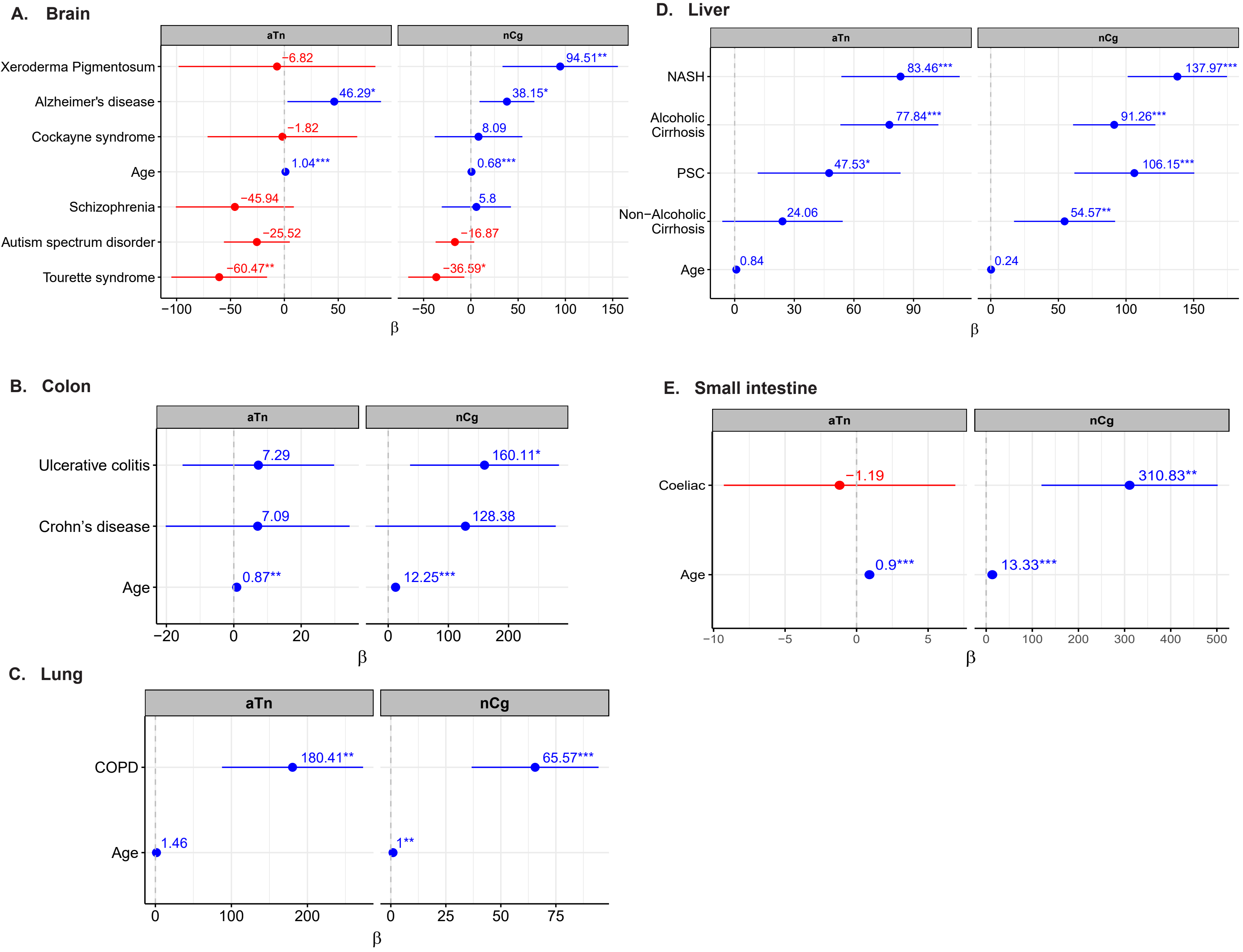
A. Brain
aTn
nCg
Xeroderma Pigmentosum
Alzheimer's disease
Cockayne syndrome
Age
Schizophrenia
Autism spectrum disorder
Tourette syndrome
−6.82
46.29*
−1.82
1.04***
−45.94
−25.52
−60.47**
94.51**
38.15*
8.09
0.68***
5.8
−16.87
−36.59*
β
B. Colon
Ulcerative colitis
Crohn's disease
Age
7.29
7.09
0.87**
160.11*
128.38
12.25***
β
C. Lung
COPD
Age
180.41**
1.46
65.57***
1**
β
D. Liver
NASH
Alcoholic Cirrhosis
PSC
Non−Alcoholic Cirrhosis
Age
83.46***
77.84***
47.53*
24.06
0.84
137.97***
91.26***
106.15***
54.57**
0.24
β
E. Small intestine
Coeliac
Age
−1.19
0.9***
310.83**
13.33***
β

Figure S10

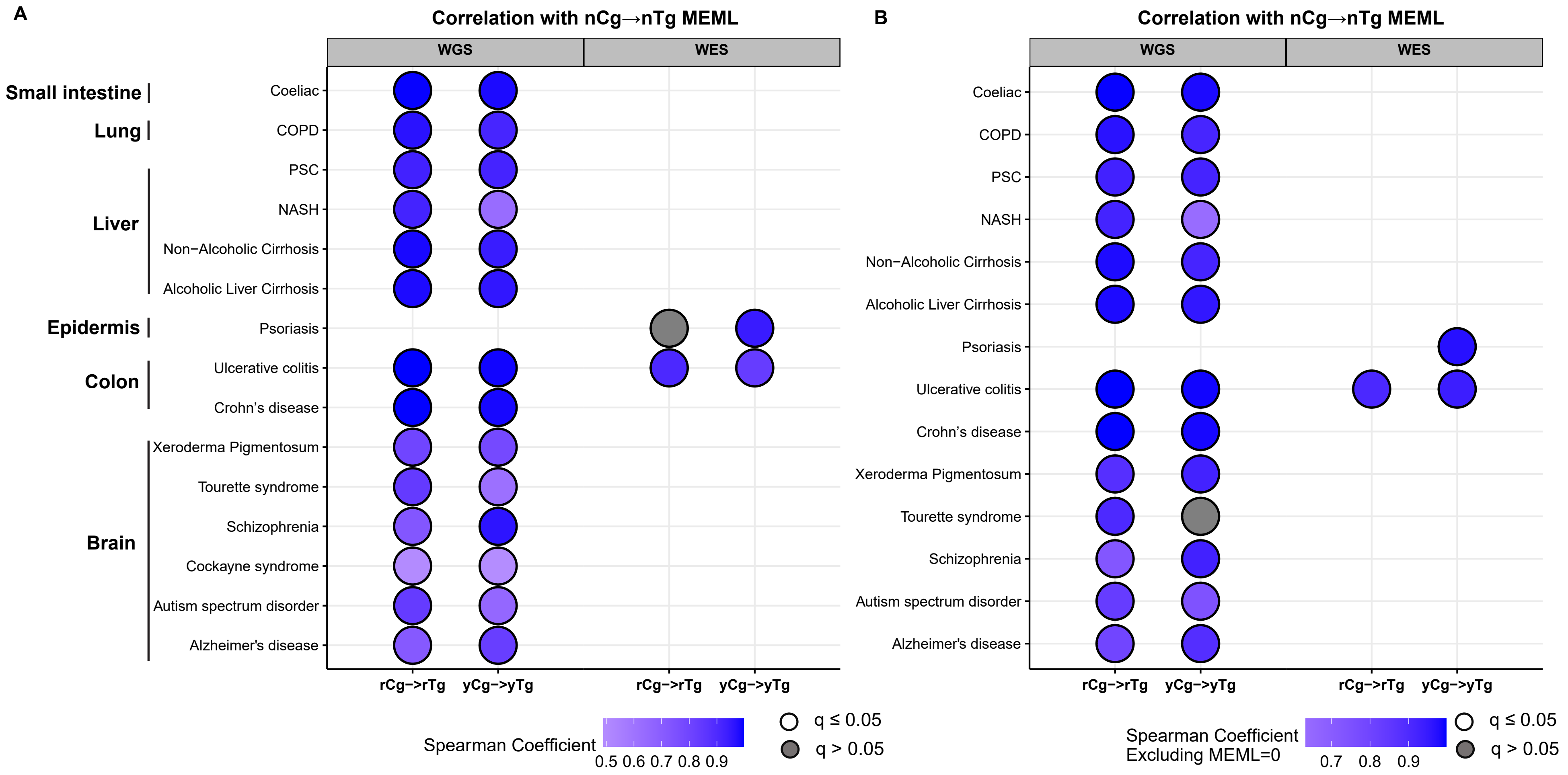
A
Correlation with nCg→nTg MEML
WGS
WES
Small intestine
Lung
Liver
Epidermis
Colon
Brain
Coeliac
COPD
PSC
NASH
Non−Alcoholic Cirrhosis
Alcoholic Liver Cirrhosis
Psoriasis
Ulcerative colitis
Crohn's disease
Xeroderma Pigmentosum
Tourette syndrome
Schizophrenia
Cockayne syndrome
Autism spectrum disorder
Alzheimer's disease
rCg−>rTg
yCg−>yTg
rCg−>rTg
yCg−>yTg
Spearman Coefficient
0.5 0.6 0.7 0.8 0.9
q ≤ 0.05
q > 0.05
B
Correlation with nCg→nTg MEML
WGS
WES
Coeliac
COPD
PSC
NASH
Non−Alcoholic Cirrhosis
Alcoholic Liver Cirrhosis
Psoriasis
Ulcerative colitis
Crohn's disease
Xeroderma Pigmentosum
Tourette syndrome
Schizophrenia
Autism spectrum disorder
Alzheimer's disease
rCg−>rTg
yCg−>yTg
rCg−>rTg
yCg−>yTg
Spearman Coefficient
Excluding MEML=0
0.7 0.8 0.9
q ≤ 0.05
q > 0.05
